# Account of Diseases in the Dispensary of the Pneumatic Institution, Dowry Square, Bristol Hotwells, from November 30, to December 31, 1801

**Published:** 1802-04

**Authors:** J. King

**Affiliations:** Bristol Hotwells


					I 401 ]
To the Editors of the Medical andPhyfical Journal.
Gentlemen, . _
X F you think the annexed lift of the Difeafes which occurred at
the Pneumatic Inftitution during laft month, worthy of a place la
your Journal, I {hall be very happy to continue it occafionally.
The gentlemen who give you fimilar lifts feem to diftar in their
Nomenclature; a greater uniformity would perhaps be defirable, if
therefore you fhould think proper to alter that which I have fol-
lowed, I lhall be thankful for your corrections, and adopt them ia
future.
Many public praftitioners may be prevented from fending you
monthly lifts, by the idea that an increafe of them would occupy
too much of the room, which would otherwife be allotted for more
important information. This objection, I think, might be removed,
by throv/ing all fuch lifts into one monthly table, of a ftandard ar-
rangement, allowing a column for the numbers of eacVReport, and
all occafional obfervations might be colleSed in a mafs, after that
table. Should this be impracticable, I hope you will excufe the
fuggeftion, as being well meant.
A detailed account of the Medical and Phyfical experiments
carried on at the Pneumatic Inftitution ill the courfe of'laft year,
comprehending extenfive trials of the muriate of lime in fcrophula,
and fome other new and little tried medicines in other important
chronic difeafes, will be publifhed in a lhort time. I am, &c.
J. KING.
Er ijlol Hot wells,
"JMl. 10, lS02.
Account cf Diseases in the Dispensary of the Pneumatic Institu-
tion, Dowry Square, Bristol Hotwells, from November 30, to
December 31, 1S01.
acute diseases.
Fever, Intermittent - 2
T-yphus - - -
Dyfentery - - - - -
Acute-Rheumatifm - -
Eryfipelas - - - - -
Small-pox - - - -
CHRONIC DISEASES.
Aft'nma ------ 4
Catarrh ------ 7
Catarrh Senil: - - - -....4
Pleurodynia - - - 2
Hasmoptde ----- 2
Phthifis Pulmon. - - - 1 z
Rhumatifm. Chron. - - 3
Scrophulous Ophthalmia - 2
Scrophulous Otitis 3
; ? Glandular Swell-
ing - - - - - - 8
Glandular Abs-
cefs ------ 4
Affe&ion of the
Joints - 4
Tabes Mefen-
terica - - ? - 3
Atrophia, from Poifon - I
Epilepfy ----- 2
Dyfpepfia -- - - - - 3
Bilious r - - 2
Hypochondriafis - - - 2
Hyiteria - - 3
Chlorofis 3
Dyfmenorrhcea
Dyfmenorrhcea
'Amenorrhea - -
Menorrhagia - -
Haemorhois - - -
Maftodynia -
Fluor Albus - -
Prolapfus Uteri
"Gonorrhoea - - -
Syphilis Primar. -
_?.? Secundaria
Dyfuria - - - -
Enurefis ------ I
Anafarca ----- 2
Afcites ------ i
Hydrocele ----- I
Impetigo ----- i
Herpes ------ 2
Petechia? fine Febre - - I
Contufion ----- i
Ulcerated Legs - - - 2
Vermes ----- 3
Inoculated for the Cow-pox 2

				

